# The Work of Faith Care: An Interpretive Phenomenological Analysis on the Role of Spirituality and Religion in Diabetes Self-Management Among Black Older Adults in the Greater Toronto Area

**DOI:** 10.1177/26350106261422687

**Published:** 2026-02-27

**Authors:** Kristina M. Kokorelias, Chukwuebuka Prince Onyekere, Hardeep Singh, Sarah E. P. Munce, Sachindri Wijekoon, Michael E. Kalu, Maurita T. Harris

**Affiliations:** Department of Medicine, Healthy Ageing and Geriatrics Program, Sinai Health and University Health Network, Toronto, Canada; Department of Occupational Science and Occupational Therapy, Temerty Faculty of Medicine, University of Toronto, Toronto, Canada; Rehabilitation Sciences Institute, Temerty Faculty of Medicine, University of Toronto, Toronto, Canada; Department of Occupational Science and Occupational Therapy, Temerty Faculty of Medicine, University of Toronto, Toronto, Canada; School of Kinesiology and Health Science, York University, Toronto, Canada; Department of Occupational Science and Occupational Therapy, Temerty Faculty of Medicine, University of Toronto, Toronto, Canada; Rehabilitation Sciences Institute, Temerty Faculty of Medicine, University of Toronto, Toronto, Canada; Rehabilitation Sciences Institute, Temerty Faculty of Medicine, University of Toronto, Toronto, Canada; School of Occupational Therapy, Western University, London, Canada; School of Kinesiology and Health Science, York University, Toronto, Canada; Faculty of Liberal Arts, Wilfrid Laurier University, Wilfrid Laurier University, Brantford, Canada

## Abstract

**Purpose:**

The purpose of the study was to explore how faith and spirituality influence diabetes self-management among older Black adults with type 2 diabetes.

**Methods:**

The researchers conducted in-depth interviews with 21 older Black adults (ages 55+) living with type 2 diabetes or prediabetes in the Greater Toronto Area. Guided by Van Manen’s interpretive phenomenology and analyzed thematically using NVivo, the researchers examined lived experiences through 4 lifeworld themes: corporeality, spatiality, relationality, and temporality.

**Results:**

Four themes emerged: faith as inner strength (spiritual resilience), sanctuaries of the soul (sacred spaces for healing), guided by faith (faith in relationships and community), and faith through time (spiritual continuity and hope). Faith helped participants manage stress, regulate emotions, sustain self-care, and strengthen social and familial bonds while providing hope across the course of illness.

**Conclusion:**

Spirituality serves as a multidimensional resource in culturally responsive, person-centered diabetes care. Integrating faith-based perspectives into interventions can foster more holistic, contextually grounded approaches that enhance well-being and long-term adherence to self-management.

Type 2 diabetes disproportionately affects Black communities, with Black older adults facing higher rates of complications, earlier disease onset, and worse outcomes compared to their White counterparts.^1,2^ The Public Health Agency of Canada reports that the prevalence of diabetes is over 2 times higher in Black Canadian adults than in White Canadians.^3^ Despite this fact, research and interventions often overlook the cultural and psychosocial dimensions that shape how Black older adults cope with living with type 2 diabetes, such as spirtuality.^4,5^

Spirituality matters because it often serves as a vital source of strength, meaning, and motivation for many Black older adults managing chronic illness. One study found that older African Americans with a stronger future time orientation and higher religiosity were more likely to consistently engage in key diabetes self-management behaviors, such as exercising, reading food labels, and checking blood glucose.^6^ These findings highlight that cultural factors, including spirituality, influence diabetes management and should be considered to reduce disparities in this high-risk population.^6^ For many Black older adults, faith and spirituality are central to understanding illness^7^ and hold promise to reducing the psychological burden of diabetes, reducing stigma, and providing culturally congruent coping techniques.^8^ Another study conducted among African American women found that they cope with chronic illness (e.g., diabetes, heart diseases, hypertension) through faith, prayer, coping, and integrating spiritual practices with conventional practices.^9^

Although terms such as “spirituality,” “religion,” and “faith” are often used interchangeably in Black literature due to their interrelated existential meaning,^10^ the study authors provide definitions here to clarify the conceptual framework. Specifically, the study teams define these terms to acknowledge their distinct theoretical roots. The study team defines *spirituality* as the individual search for the sacred or meaning in life, *religion* as the pursuit of significance within institutional or communal structures designed to facilitate spirituality, and *faith* as belief or trust in something that may not be directly observable or proven, often centered on religious or spiritual convictions.^11^ However, because these concepts are deeply intertwined in many Black communities and are often experienced as a unified set of beliefs and practices, the researchers later consider them collectively when discussing their role in chronic illness management.

In this approach, Black individuals structure their approach in different ways, such as seeing God as a helper who is “in the background,” “in the forefront,” or as “healer,” which can shape motivation and daily health-seeking behaviors that enable them to manage their diabetes.^12^ Prayer, church attendance, Bible reading, and media-based spiritual activities are frequently used coping strategies and resilience tools that aid diabetes management.^13^ These practices foster hope, motivation, strength, and a positive mindset, which are conducive to healthier diabetes self-care.^13^ From a spiritual perspective, faith-based approaches and spiritual counseling have been incorporated into diabetes education programs, positively affecting knowledge, empowerment, and self-efficacy for change among Black participants. Prior studies on spirituality and diabetes self-care have largely focused on African Americans in the United States. A study focused on Black Canadians is warranted because historical experiences, migratory backgrounds, and cultural diversity shape faith-based caregiving differently and Canada’s social and health care contexts may limit the transferability of US findings. Existing research also largely excludes non-African American Black subgroups, highlighting a gap and the need for culturally responsive interventions that are specific to the Canadian health care context.^14^ Swaleh and Yu^14^ explicitly note that “research on diabetes self-management in [the Black] community is lacking” in Canada.

Despite the centrality of spirituality to illness management, spiritual dimensions are rarely integrated into clinical care or diabetes education programs.^15^ Diabetes education programs that integrate spirituality often treat it as an add-on to biomedical care rather than as a mode of meaning-making and mutual attunement that shapes motivation, trust, and adherence.^16^ Although community-based interventions delivered through Black-led faith organizations (eg, churches) have shown promise for improving knowledge and self-management behaviors, few have explored how older individuals themselves use faith every day to navigate the practical demands of diabetes care.^17,18^ Church-led programs tend to emphasize education and peer support, but they rarely examine the lived experience of spiritual coping, meaning-making, and internal motivation.^17,18^ Integrating spiritual coping into clinical practice is directly relevant to self-management because it influences how patients understand, engage with, and sustain their own care. When practitioners assess and respond to patients’ values, beliefs, and social networks, they support patients in making informed choices, setting personally meaningful goals, and drawing on their own coping strategies. This alignment strengthens patients’ ability to manage symptoms, adhere to treatments, and navigate the emotional and social aspects of their condition, making self-management more holistic and effective. Furthermore, most diabetes-spirituality studies mix ages or focus on younger adults. Addressing this gap is especially important given the growing recognition that effective diabetes care must be person-centered and attuned to patients’ lived experiences, including their values and worldviews.^19^

To the researchers’ knowledge, few in-depth qualitative studies center the lived spiritual practices of Black older adults managing prediabetes and type 2 diabetes in Canada, and existing research often pools ages, limiting generational specificity.^20^ There is also a limited understanding of how the role of spirituality in self-management may shift over different stages of diabetes or with changes in life circumstances.^20^ Interventions implemented in community settings tend to emphasize education and peer support. Yet they do not typically document how spiritual meaning-making is enacted in daily self-management or translate that knowledge into culturally grounded clinical pathways. Understanding how spiritual meaning-making is enacted in daily self-management can inform interventions that truly support patients’ routines, coping strategies, and decision-making in ways that align with their values. This study addresses these gaps by using an interpretative phenomenological approach to center Black older adults’ own accounts of the “spiritual work” of diabetes self-management.^21^

## Methods

### Study Design

Understanding lived experiences requires attention to how chronic illness is lived and experienced in everyday life. Van Manen’s interpretive phenomenology provides a framework for examining these experiences through 4 fundamental lifeworld themes: lived body (corporeality), lived space (spatiality), lived human relations (relationality), and lived time (temporality).^22^ Lived body captures how individuals experience their physical health, bodily sensations, and limitations in managing illness. Lived space reflects the influence of environments, such as the home, church, or community, on daily practices and spiritual coping mechanisms. Lived human relations emphasizes the significance of social and spiritual connections, including family, peers, and God, in shaping motivation and support.^22^ Lived time considers how past experiences, present circumstances, and future expectations influence understanding of illness, decision-making, and meaning-making.^22^

This study employed an interpretative phenomenological approach, informed by the guidelines of Van Manen.^22^ Interpretative phenomenology seeks to understand the meanings of human experiences, emphasizing the body as historically situated, relational, and inseparable from the world.^22^ Phenomenology describes all experience as perceptual, integrating mind, body, world, and time, with embodiment giving meaning to life’s temporal, spatial, and relational dimensions.^23^ This program of research was approved by the University of Toronto Research Ethics Board (REB No. 00047368).

### Sampling and Recruitment

This study purposively sampled a diverse group of English-speaking Black adults ages 55 and older, including both Canadian-born and immigrant participants living in suburban Ontario.

Potential participants were invited via email, and recruitment materials were distributed through Black-led community organizations, religious institutions, local health and social service agencies, ethnic newspapers, community bulletins, Afro-Caribbean grocery shops, social media networks, and community-organized diabetes events. Recruitment materials included study details and the study team’s contact information. Interested individuals were asked to contact the researchers for eligibility screening. Participants were selected based on age (55 and older), self-identification as Black, English literacy, living in the Greater Toronto Area (GTA), and the ability to reflect on and verbally share their lived experiences with prediabetes and type 2 diabetes.

Exclusion criteria included being under 55 years of age, cognitively impaired, institutionalized, or non-English speakers or residing outside the GTA. To avoid conflicts of interest, no participants were known to the research team. Of 41 potential participants screened, a purposive sample of 21 participants ages 55 to 83 was selected, including 12 females and 9 males. Five participants had prediabetes, and 16 had type 2 diabetes (see Table 1 for participant demographics). All participants provided written consent prior to the interview and verbal consent at the start of the interview.

**Table 1.** Participant Sociodemographic Characteristics (n = 21)

Variable	Categories	Frequency (%)
Age	55-64	11 (52.4%)
	65-74	8 (38.1%)
	75-84	2 (9.5%)
Gender	Female	12 (57.1%)
	Male	9 (42.9%)
Ethnicity	Caribbean	10 (47.6%)
	African	4 (19%)
	Black African	6 (28.6%)
	Black Caribbean	6 (28.6%)
	Black Canadian	4 (19%)
Immigration status	Immigrant	18 (85.7%)
	Canadian-born	0 (0%)
	Not reported	3 (14.3%)
Language	English	21 (100%)
	Patois	10 (47.6%)
	Igbo	3 (14.3%)
	Yoruba	1 (4.8%)
Diagnosis	Diabetes	16 (76.2%)
	Prediabetes	5 (23.8%)
Duration of diagnosis, y	1-5	10 (47.6%)
	6-10	5 (23.8%)
	>10	6 (28.6%)
Current treatment/management approach	Lifestyle changes (diet, exercise)	19 (90.5%)
	Oral medications	14 (66.7%)
	Insulin	5 (23.8%)
	Regular monitoring of blood glucose levels	15 (71.4%)
Household composition	Living with a spouse/partner	11 (52.4%)
	Living with children	6 (28.6%)
	Living alone	5 (23.8%)
	Living with other family members	1 (4.8%)
Role of family in diabetes management	Actively involved	11 (52.4%)
	Occasionally involved	5 (23.8%)
	Not involved	5 (23.8%)
Previous participation in any diabetes self-management programs or interventions?	Yes	4 (19.05%)
	No	17 (80.95%)
Primary sources of information or support for managing diabetes?	Health care providers	19 (90.5%)
	Family and friends	10 (47.6%)
	Community organizations	3 (14.3%)
	Online sources	14 (66.7%)
	Support groups	3 (14.3%)

### Data Collection

Each participant was invited to participate in an in-depth, one-on-one interview lasting between 45 and 60 minutes. Interviews were audio-recorded and designed to elicit rich descriptions of participants’ experiences with prediabetes or type 2 diabetes, including the role of spirituality in self-care management. The interview guide was cocreated by the research team and a Black older adult living with type 2 diabetes (trained peer researcher). The interview guide included open-ended questions, but participants were encouraged to focus on aspects of their experience most meaningful to them, allowing the data to reflect their lived realities rather than being limited to predetermined questions. Participants were given the option of being interviewed by the trained peer researcher or a Black, male, PhD-student research assistant trained in qualitative research. Interviews were scheduled at times convenient for each participant and conducted either in person, by Zoom, or by phone. Participants selected the method of participation where they felt most comfortable.

### Data Analysis

Verbatim transcripts the researchers reanalyzed alongside reflective journaling by the interviewers and the first author helped clarify assumptions, preunderstandings, and insights into participants’ experiences. This reflective process enhanced rigor by making the researchers’ perspectives and potential assumptions explicit based on their prior experience working with older adults who manage chronic conditions.

Following Van Manen’s approach to analysis, the researchers’ analysis focused on four lifeworld themes—lived body (corporeality), lived space (spatiality), lived human relations (relationality), and lived time (temporality)—to guide reflection on participants’ lived experiences.^22^ This approach included orienting to the phenomenon and explicating assumptions, investigating experiences as lived rather than conceptualized, conducting thematic analysis through reflective interpretation, describing the phenomenon through iterative writing, maintaining focus on the central research question, and balancing consideration of individual and overall themes.^22^ Transcripts were read multiple times to ensure immersion in the data. The researchers referred to the participant demographics while reading the transcripts to contextualize the interviews based off sex. A framework method of charting was used to organize emergent codes and themes in a matrix, allowing for comparison both within and across participant accounts. First, each transcript was examined for recurring and meaningful concepts related to spirituality, religion, and diabetes self-management by 2 researchers (KMK and CPO) independently. Coded concepts were organized into individual tables, which were then collapsed into broader categories, such as “emotional coping” or “faith-based practices.” Across the data set, codes with similar conceptual meanings were combined to generate overarching themes, which the researchers re-refined iteratively and visually organized in analytical charts to depict patterns and relationships. These steps facilitated a deeper understanding of participants’ lived experiences of spirituality and religion, their relational context to diabetes, and the meaning of spirituality and religion in their daily self-management.^24^ To enhance the trustworthiness of the findings, the research team engaged in reflective journaling throughout the analysis process and conducted member checks with participants to validate interpretations of the data.^25^

## Results

**Figure 1.**
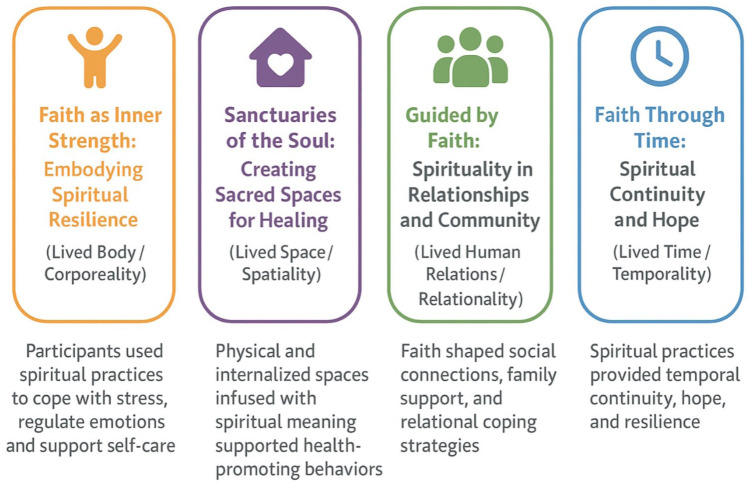
Four dimensions of religion and spirituality in living with diabetes.

In this study’s analysis, the researchers identified 4 key themes that illustrate how faith and spirituality intersect with participants’ experiences of managing diabetes in positive ways (Figure 1). The first theme, “faith as inner strength: embodying spiritual resilience (lived body/corporeality),” highlights how participants used spiritual practices to cope with stress, regulate emotions, and support self-care. The second theme, “sanctuaries of the soul: creating sacred spaces for healing (lived space/spatiality),” reflects how physical and internalized spaces infused with spiritual meaning supported health-promoting behaviors. The third theme, “guided by faith: spirituality in relationships and community (lived human relations/relationality),” emphasizes the role of faith in shaping social connections, family support, and relational coping strategies. The fourth theme, “faith through time: spiritual continuity and hope (lived time/temporality),” illustrates how spiritual practices provided temporal continuity, hope, and resilience across participants’ long-term experiences with diabetes. Each theme captures a distinct dimension of lived experience (i.e., bodily, spatial, relational, and temporal) through which faith and spirituality influence diabetes self-management. Although the researchers acknowledge the delineation of spirituality (the individual search for meaning), faith (beliefs), and religion (organized practices and institutions), this study’s analysis treated them as a single, fluid concept. This decision was driven by how participants spoke; spiritual and religious language were used interchangeably, and the practices they described often combined private meaning-making with communal worship. For many participants in the study sample, spiritual identity and religious practice are deeply entangled, making a strict analytic separation both artificial and inconsistent with their lived experience. As such, the researchers followed their own categories rather than imposing external conceptual boundaries. Each theme is described in more detail in the following, with illustrative quotes sourced by participant sex and age.

### Faith as Inner Strength: Embodying Spiritual Resilience (Lived Body/Corporeality)

Participants described their faith and spirituality as deeply connected to their bodily experiences, serving as a source of strength to manage both the psychological and physical challenges of living with diabetes. Faith, whether religious or spiritual, became a flexible, participant-specific resource for navigating the psychological and physical challenges of diabetes. Participants highlighted how faith provides a calming effect and helps them to regulate stress. Many participants drew strength from organized religious practices, such as prayer, worship music, and reciting Bible verses, reflecting faith grounded in institutional religion. Some participants described a more personal, internalized sense of spirituality, which offered meaning, hope, and self-regulation outside formal religious structures. One participant said,

Yes, I would say yes, my faith helps me to manage stress. . . . I calm myself down . . . I try to remember a Bible verse that I can read or recite in my head that will calm me down . . . I think my religion and my faith helps me to cope with my diabetes. (P001, female)

Another participant echoed, “Okay, my faith and my religion, it’s something that is really helping me to cope with stress. For instance, like a Christian, I do always try to study the Bible each time I’m alone” (P020, male). Together, these quotes illustrate how religious beliefs and structured practices can become an anchor during stressful or overwhelming moments.

Similarly, participants often spoke about the motivational and restorative role of prayer and worship music, explaining that these practices support their self-management by providing the emotional strength needed to adhere to daily diabetes care routines, such as physical activity. One participant said, “I am a devoted Christian. I pray a lot, and also, I worship a lot. . . . Listening to worship music . . . gives me the motivation, the self-motivation I need to manage stress . . . that really helps me a lot to manage stress” (P016, male). This connection between support and practical self-management highlights how faith-based coping extends beyond comfort to tangible health-related action.

### Sanctuaries of the Soul: Creating Sacred Spaces for Healing (Lived Space/Spatiality)

Participants described how religion and spirituality shaped the ways they experienced and inhabited both physical and symbolic spaces, creating environments that directly supported diabetes self-management. Surprisingly, participants described creating sacred spaces for healing outside traditional religious structures. For instance, P028 highlighted how everyday activities could be infused with spiritual meaning, creating an internal sacred space through prayer and music that supported coping and diabetes self-management. These sacred places became sanctuaries for reflection, stress reduction, and purposeful engagement in health behaviors, such as exercise. As participant P028 (male) described, “My religion . . . I just prayed and did [exercises] for God . . . going out of the house or listening to music like I always did.” Some of these spaces are intentionally created for place to promote several health behaviors, such as monitoring blood glucose, preparing healthy meals, or maintaining exercise routines.

Some participants drew on organized religious spaces, such as churches or home rituals, to reinforce structured practices and a sense of accountability in managing their condition. Others created internalized or symbolic spiritual spaces through prayer, meditation, or listening to music, which helped them focus, regulate emotions, and maintain motivation for daily diabetes care.

For example, P023 (female) described her home as spiritually enriched, reflecting both religious and personal significance, as a place of celebration, reflection, and spiritual grounding:

When my stress level is high, I get headaches and then that’s when I know my blood sugar is high. So I usually go for a brisk walk . . . or just go somewhere and just sit quietly, I meditate. I pray . . . I just pray, ask God to guide me in dealing with it.

### Guided by Faith: Spirituality and Religion in Relationships and Community (Lived Human Relations/Relationality)

Participants described how faith and spirituality shaped their social relationships in ways that directly influenced their diabetes self-management. Religion provided guidance for how they interacted with family, shared responsibilities, and sought or offered support around the practical demands of living with diabetes, such as preparing healthy meals, remembering medications, or managing medical appointments. Spirituality offered relational tools that helped participants navigate stress, reinforce cultural values, and maintain accountability within their social networks, which in turn supported adherence to diabetes care routines.

A participant described how songs have formed a core aspect of their family bond and in turn provide him with the strength to manage diabetes:

So, my family, there’s a special song I have, that is composed in the house by my children for me. My family do sing that song for me every evening. So it’s a kind of always been helpful for me and then I love it so much. (P020, male)

One described teaching her daughters about relying on both God and family support, highlighting how spiritual values informed the sharing of caregiving responsibilities and coping strategies that are essential for managing diabetes: “I tried to tell my daughters . . . ‘Look, you have a family, God first, and then your family . . . ask somebody to help you . . . I cannot do this all by myself. I need help’” (P001, female).

Another participant described how their faith influenced their understanding of family patterns and motivated proactive engagement with health behaviors, including her approach to managing diabetes and preventing illness in herself and others: “I saw the pattern . . . so I started talking to God about diabetes. I can see through it, but all is well” (P026, female).

### Faith Through Time: Spiritual Continuity and Hope (Lived Time/Temporality)

Participants drew on spirituality and religion to create a sense of continuity and hope over the long-term course of living with diabetes. Rather than seeing diabetes management as a series of isolated tasks, participants framed it within a temporal narrative shaped by faith. The study team examined whether participants who had prediabetes, were newly diagnosed, or had long-standing diabetes differed in how they framed diabetes management within a temporal narrative shaped by faith, but the researchers did not observe notable differences. Spiritual practices such as prayer, reflection, and trusting in God helped them connect past experiences, present challenges, and future expectations, giving meaning to the ongoing work of managing their health: “My faith plays a crucial role. I find comfort in praying, reading the Bible, and trusting that it’s not the end. God helps me manage it” (P019, female).

A participant emphasized the stabilizing role of decades-long faith, describing how her long-standing spiritual beliefs allowed her to approach diabetes with confidence and resilience:

Oh. I always believe we are Christian. And I’ve been faithful for more than 40 years. I always believed that there is nothing that He [God] cannot do. So, we put it to God about the diabetes things, sincerely, it does not bother me really. Because the moment I said, “Oh God,” I pray about it, and I believe that it can be solved. So it’s just a factor. (P026, female)

Participants also illustrated how integrating spiritual practice into daily routines provided practical and psychological support for ongoing diabetes management: “Thanks to the clinic . . . I asked God, I asked Him . . . help with this diabetes. . . . And now I really just do that” (P028, male).

## Discussion

Spiritual health is a critical yet often overlooked component of overall health that shapes how individuals experience and manage illness.^26^ The researchers conducted this study to understand how Black older adults in Canada use spirituality and religion in their daily management of prediabetes and type 2 diabetes, addressing gaps in culturally grounded, person-centered care and the limited recognition of spiritual practices in clinical interventions. The researchers identified 4 interrelated themes, with religion and spirituality as the foundational antecedent of faith and as intermediate beliefs that enable Black older adults to manage diabetes. These themes show that faith, grounded in religion or spirituality, manifests as corporeality (the lived body) within spatial sanctuaries of lived space expressed through relational experiences (lived relationships) across multiple time periods (lived time). These results align with Van Manen’s lifeworld existential, which provided the analytic lens for this study.^22^ Taken together, the study’s themes suggest that faith and its antecedents (spirituality and religion) function as an integrated, multidimensional resource for managing type 2 diabetes and prediabetes. They not only are a personal source of emotional and bodily resilience but also shape how participants experience their environments, connect with others, and make sense of their illness over time. The findings of this study highlight the importance of integrating faith expressed through religion and spirituality into diabetes care for Black older adults. Clinicians can support patient-centered care by recognizing spiritual and religious practices as meaningful resources that foster resilience, motivation, and adherence to self-management routines. This could include asking patients about spiritual beliefs during assessments, collaborating with faith leaders or community organizations, and providing space for prayer, meditation, or faith-based music in clinical and community settings. Incorporating spiritual dimensions into care plans may enhance engagement, strengthen family and community support networks, and improve the overall relevance and cultural responsiveness of diabetes interventions for this population.^27,28^

This study’s findings align with prior studies showing that spirituality supports psychological resilience and stress regulation among older adults with chronic illness.^29,30^ However, the study’s findings extend this work by emphasizing the direct link between spiritual and religious practices and daily diabetes self-management tasks, such as exercise and stress management. Participants in this study described faith as integral to sustaining motivation for routine health behaviors. Similar to other studies with Black adults living with diabetes, participants in the study’s study described a direct relationship with God as central to managing diabetes, emphasizing prayer, trust, and reliance on faith to cope with the daily burden of the illness.^12^ However, the study’s findings highlight broader dimensions that demonstrate how spirituality permeates not only personal coping but also spaces, relationships, and long-term illness trajectories. Echoing Duke,^31^ the study’s findings confirm that spirituality provides motivation, hope, and a framework for assuming responsibility in the day-to-day management of diabetes. Although the study’s findings and the existing literature highlight the agency of patients in navigating self-management beyond biomedical guidance,^31^ it situates spirituality in a more collective and contextualized frame, showing how faith informs family interactions and caregiving responsibilities. Lastly, in line with an existing structured review, the study’s findings support the notion that spiritual practices are deeply integrated into self-care routines, shaping how individuals interpret illness and engage in health behaviors.^15^ Yet the study’s findings extend this by showing concrete examples of how Black older adults transform everyday environments (e.g., homes, music, plants) into sacred spaces that actively support self-management, a nuance not fully captured in the review.^15^ Although relying on spiritual beliefs and family support can provide emotional resilience, guidance, and motivation in managing chronic illness, it may also create tension if patients feel overly dependent on external success, potentially limiting self-efficacy. A study of multiple myeloma (cancer) patients found that “negative religious coping” (eg, feeling punished by God, feeling abandoned) was associated with poorer physical functioning and more distress, pain, and fatigue.^32^ This juxtaposition highlights the balance between community- or faith-based support and fostering personal agency, suggesting that self-management programs could complement these supports by empowering patients to take active, informed roles in their daily care.

Participants emphasized the role of faith in shaping family responsibilities, caregiving practices, and relational accountability. This theme echoes literature on communal coping and the importance of relational networks in diabetes care.^33^ However, the study’s findings extend this literature, suggesting that relational support in Black older adult communities has spiritual underpinnings. These findings could inform practice by framing health education and interventions in ways that align with patients’ spiritual values, collaborating with faith leaders and churches as trusted partners, and creating culturally responsive care strategies that honor the role of spirituality in fostering resilience, accountability, and collective caregiving. Pragmatically, this can be done by embedding caregiver support groups in collaboration with churches or faith-based organizations and training health care providers to respectfully incorporate patients’ spiritual practices into care planning. Programs might also create flexible spaces for prayer, meditation, or faith-based music within clinical or community settings as family engagement strategies.

Although none of the 4 themes could be transferred to patient populations outside of older adults, the accounts were deeply shaped by participants’ experiences of growing older with diabetes. For example, references to long-standing faith practices and the importance of intergenerational family teaching suggest that spirituality was experienced as something cumulative, layered, and transmitted across the life course rather than confined to the present. However, the study did not explicitly take a life course perspective, which might have illuminated how participants’ spirituality and self-management strategies were shaped by earlier life experiences (eg, migration histories, exposure to systemic racism, cultural traditions of religious practice) and how these intersect with aging in later life.^34^ The life course perspective is a framework in health research that views health and disease as the result of cumulative biological, behavioral, and social influences across an individual’s lifetime.^34^ Although prior research with non-Black populations has also documented the role of spirituality in fostering hope among patients with diabetes,^35^ our findings extend this perspective by showing how temporal continuity of faith and religion supports the long-term self-management in diabetes. A life course lens could have also revealed how turning points, such as diabetes diagnosis, retirement, or loss of loved ones, influenced participants’ reliance on spirituality for resilience. For example, for clinical practice, health care providers may consider asking patients whether they have previously found support through spirituality or faith-based resources to help them navigate life’s challenges. Such discussions could facilitate more personalized care and support patients’ self-management strategies, particularly for those managing chronic conditions. In addition, future research is needed to investigate how the role of faith in diabetes self-management evolves across different stages of life and how older and oldest-old populations may engage spirituality differently. Such work could clarify whether the patterns observed here, such as creating sacred home spaces or integrating faith into daily routines, are amplified with age.

### Limitations

This study has several limitations. All participants had to speak English. The English-only requirement limits the generalizability of findings, potentially excluding non-English-speaking populations whose experiences with self-management and spiritual coping may differ. Moreover, the study was limited to a geographically and linguistically narrow sample that may not reflect the diversity of Black older adults across Canada. The cross-sectional qualitative design could not capture how spirituality in diabetes self-management evolves over time. Potential interviewer influence may have shaped how participants shared their experiences, particularly given the sensitive and personal nature of spirituality, where participants might have emphasized or downplayed certain practices given the interviewer’s identity or perceived expectations. The researchers acknowledge the limitations of our analytic technique of merging spirituality and religion. The researchers’ analysis approach may have reduced conceptual precision. Because the secondary analysis was not designed to probe differences between personal spirituality and organized religion, the researchers could not reliably separate how each uniquely influenced self-management. Future work should include purposive questions and validated measures that distinguish personal spiritual beliefs from formal religious practices and consider mixed-methods or longitudinal designs to unpack how these dimensions differentially affect behavior and outcomes. Finally, because this study did not directly connect participants’ spiritual practices to clinical outcomes, such as glycemic control, medication adherence, or reduced diabetes-related complications, the implications for improving biomedical indicators of health remain uncertain. Future research could explore how spiritual coping influences concrete self-management behaviors and clinical outcomes, the mechanisms linking faith and health, and ways to integrate spiritual meaning-making into culturally sensitive interventions. This limits the ability to transfer findings to broader clinical populations and emphasizes the need for mixed-methods or longitudinal research to establish how spiritual coping influences not only subjective well-being but also measurable health outcomes.

## Conclusion

This study highlights the central role of spirituality and religion in the daily self-management of diabetes among Black older adults in Canada. Through the lens of Van Manen’s lifeworld themes, participants’ experiences revealed that faith shapes not only emotional resilience and motivation but also the ways individuals inhabit physical spaces, engage with social networks, and navigate long-term illness trajectories. Spiritual practices were deeply integrated into everyday routines, providing meaning, hope, and continuity across time and influencing both personal and relational approaches to care. These findings highlight the importance of recognizing spirituality as a multidimensional resource in culturally responsive, person-centered diabetes care. Integrating spiritual considerations into interventions and clinical practice could support more holistic, contextually grounded approaches to diabetes management, ultimately enhancing both well-being and adherence to self-care.
